# A Real-Time Automatic Plate Recognition System Based on Optical Character Recognition and Wireless Sensor Networks for ITS

**DOI:** 10.3390/s20010055

**Published:** 2019-12-20

**Authors:** Nicole do Vale Dalarmelina, Marcio Andrey Teixeira, Rodolfo I. Meneguette

**Affiliations:** Department of Informatics, Federal Institute of São Paulo, Catanduva 15808-305, Brazil; marcio.andrey@ifsp.edu.br (M.A.T.); meneguette@ifsp.edu.br (R.I.M.)

**Keywords:** intelligent transportation system, automatic license plate recognition, wireless sensors networks, optical character recognition

## Abstract

Automatic License Plate Recognition has been a recurrent research topic due to the increasing number of cameras available in cities, where most of them, if not all, are connected to the Internet. The video traffic generated by the cameras can be analyzed to provide useful insights for the transportation segment. This paper presents the development of an intelligent vehicle identification system based on optical character recognition (OCR) method to be used on intelligent transportation systems. The proposed system makes use of an intelligent parking system named Smart Parking Service (SPANS), which is used to manage public or private spaces. Using computer vision techniques, the SPANS system is used to detect if the parking slots are available or not. The proposed system makes use of SPANS framework to capture images of the parking spaces and identifies the license plate number of the vehicles that are moving around the parking as well as parked in the parking slots. The recognition of the license plate is made in real-time, and the performance of the proposed system is evaluated in real-time.

## 1. Introduction

The number of motor vehicles is growing in most places of the world at an alarming rate, causing big issues like traffic congestion, low quality of air and so on. According to McKinsey [1], it is expected that the number of vehicles will increase to 1.32 billion units by 2020. On the other hand, the development of Intelligent Transportation Systems (ITSs) is becoming a feasible solution for many current issues. ITS is the junction of various technologies that focus on service and application delivery that will monitor and manage the transportation system, making it more comfortable and secure [2].

Wireless Sensor Network (WSN) is one example of technology that has been used to build ITS solutions. WSN has received considerable attention from researchers, governments and businesses due to its potential to provide easy and cost-effective solutions in many different areas [3]. Moreover, WSN can be integrated with other technologies to assist ITS services [4]. Automatic License Plate Recognition (ALPR) is an example of ITS service that can be built using WSN technologies. 

ALPR has become a very important tool in the ITS field because this service helps in the monitoring and control of the vehicles. However, due to the diversity of plate formats (for example plate size, plate background, character size, plate texture and so on), the accurate development of an ALPR system is a challenging task, mainly when it comes to an open environment, where there are variations in the illumination conditions during image capturing [5].

According to Patel et al. [6], the development of a conventional ALPR system can be divided into four steps: (1) vehicle capture image, (2) number plate detection, (3) character segmentation, and character recognition. The main functions of steps (1) and (2) are to detect the object (vehicle) and capture the image to find out the potential regions within the image that may contain the license plate. The discussion of the object detection algorithms is beyond the scope of this paper. However, we refer the reader to notable works in [7,8] for detailed technical discussions of these algorithms. Regarding step (3), the main function is to isolate the foreground characters from the background within the detected license plate region. In step (4), methods are employed to try recognize the characters.

The ALPR system can have two variations: on-line and off-line. As described in [9], in the online ALPR system, the localization and interpretation of license plates take place instantaneously from the incoming video frames, enabling real-time tracking of moving vehicles through the surveillance camera. On the other hand, an offline ALPR system captures the vehicle images and stores them in a centralized data server for further processing.

Based on the characteristics above described, several methods have been proposed for ALPR systems, where each one has its advantages and disadvantages [10,11]. Most works of ALPR system [12,13], use the edge proprieties as features for localizing the license plate regions, and some of these works [14] capture the image of a vehicle carefully placed in front of the camera taking a clear image of the license plate. However, in a practical scenario, there may be huge variations in lighting conditions that make plate recognition even more difficult. Even having several works about the ALPR system, the development of an ALPR system that can work out accurately on open environments with variations on the illuminations is still necessary. 

An intelligent parking service based on wireless sensors for managing public or private spaces named Smart Parking Service (SPANS) has been developed by [15]. The SPANS system is a framework built using low-cost sensors coupled in a node to monitor the parking spaces. The nodes capture the images of the parking spaces and periodically send them to the intelligent transport system. The system provides mobile access in which the user can perform various management functions, such as finding vacant parking lots and a statistical report.

In this paper, a vehicular identification system based on OCR and WSN for ITS is proposed. The proposed system is implemented as a new functionality inside of the SPANS system described in [15]. The system has access to the images from the camera and uses an OCR to detect the license plate number of the vehicles that are moving around the parking as well as parked in the parking slots. Once a new vehicle is detected, the proposed system detects the region of the plate and applies an algorithm to extract the image of the plate. This image is processed and an OCR method is used to extract the characters and numbers that compose the license plate. This information is stored on a cloud and it can be used, for example, in the department of transportation to detect stolen vehicles or issues in the vehicle’s documentation. The performance of the proposed algorithm to recognize the license plate characters is analyzed in an open environment where the SPAN system is installed. So, the experiments are carried out in a real environment in real-time. The main contribution of the paper is the development of the ALPR system to be used in an open environment as well as the evaluation of the Tesseract 4.0 in real time in an open environment. The proposed system is evaluated in a real environment in real-time.

The remainder of the paper is organized as follows: Section 2 presents a brief background of the SPANS system, the OCR, and the related works. Section 3 describes the proposed system. Section 4 presents the scenario and the performance measurements. Section 5 discusses our results. Finally, Section 6 concludes the paper with a summary of the main points and outcomes. 

## 2. Background

In this section, we briefly present a description of the SPANS Parking Service used as our framework to insert the proposed system as well as the related works.

### 2.1. The SPANS Parking Service Framework

**Figure 1:**
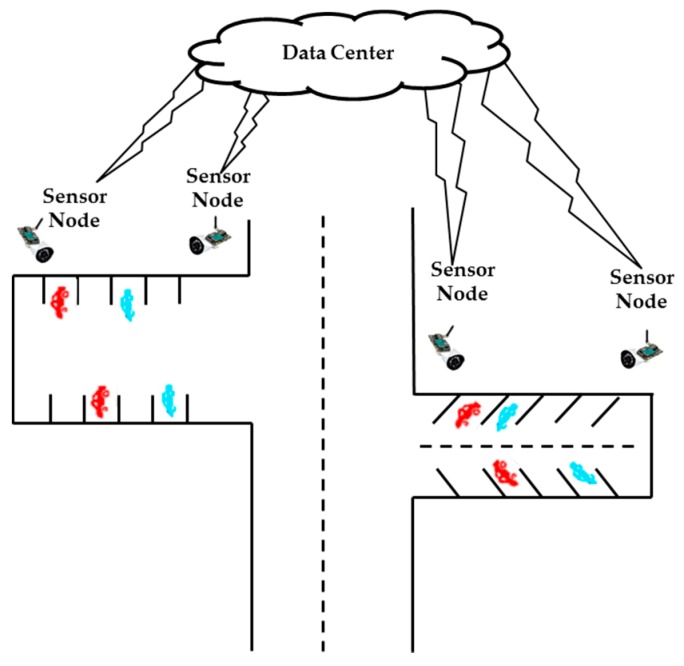
The infrastructure used by SPANS [5].

The SPANS is a framework used to detect available parking slots. This service is integrated with an intelligent transportation architecture that provides the structure necessary to detect, manage and notify the vehicles about the space. The SPANS infrastructure uses sensors to detect parking slots and cameras to record activities around the parking spaces. Sensors and cameras are used to detect significant parking information so that it is possible to verify if there are any spaces available. The infrastructure consists of a data center (cloud) which provides mechanisms for data abstraction and data processing, as well as communication mechanisms between drivers and sensors. Figure 1 shows an abstraction of the SPANS infrastructure that is used to detect and notify users about free parking.

As can be seen in Figure 1, the data center has sensors that detect changes in the environment and receive information about the parking slots. This information is processed by the SPANS that will detect, using computer vision techniques [16], whether the parking slots are available or not. After the image processing, the system will provide information for the users about the available slots through a driver application.

#### 2.1.1. The Data Center

The data center receives information (images) from sensors and sends information to mobile applications. This is done through a web RESTful (Representational state transfer) service which is implemented in the data center. So, images are captured by the sensors, processed, and the information about the availability of the parking slots are sent to the mobile applications. All data collected by the sensors are processed, analyzed and stored in a database that is accessible from the mobile application.

The wireless sensor node is based on a Raspberry Pi [17] model B. Raspberry Pi has a USB wireless network interface and a traditional webcam. Raspberry uses the Raspbian operating system, a Python program to read data from the GPIO (General Purpose Input/Output) pins and a program to monitor the webcam video signals. When a movement is detected, the web can wait a few seconds and take a picture of the vehicle that is arriving or leaving the parking slot. 

#### 2.1.2. The Parking Slots Information

**Figure 2:**
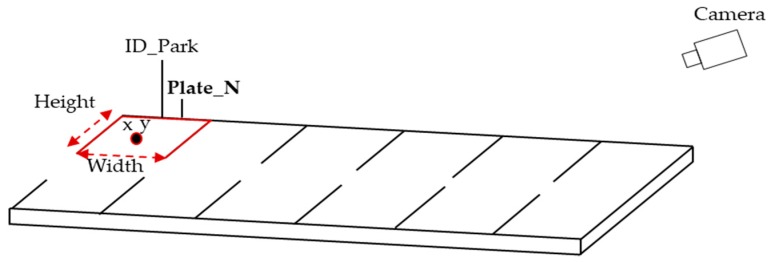
Parking information.

The parking area is divided into several spaces (slots), as shown in Figure 2. Originally, each parking space has the following information:

- Park_Id: Parking space identification;
- Desc_park: Description of the parking space;
- *X*_coor: Coordinate “*X*” of the parking space;
- *Y*_coor: “*Y*” coordinate of parking space in parking areas;
- Width: Width of parking space;
- Height: Height of the parking space;
- Status: Stores the current status of the parking space. 0 is available; 1 is busy;
- Plate_N: Stores the plate license information.

In this work, we inserted new information named “Plate_N” in the SPANS framework. So now, the information detected by our system is stored in the SPAN system and all information about the parking is sent to a database. Once the parking spaces have been defined, using a camera, the system receives an image called “Base Image” of each parking space, taking into account the *X* and *Y* coordinates and the width and height values. The “Base Image” is stored in the database and it is used to check if the parking space is available or occupied. The details about the SPAN service framework can be found in [15].

### 2.2. Optical Character Recognition

Optical Character Recognition (OCR) is one of the most widely studied problems in the field of pattern recognition and computer vision [18]. Despite being widely studied, OCR remains a challenging problem when used in unconstrained environments like parking areas. Moreover, it is necessary for improving the recognition rate on ambiguous characters, such as (B-8), (O-0), (I-1), (A-4), (C-G), (D-O), (K-X), and broken characters [19]. Several studies about the performance of the OCR are made using a dataset of images. In this paper, the OCR used is the Tesseract 4.0 [20]. The version 4.0 of the Tesseract implements a Long Short-Term Memory (LSTM)-based recognition engine which is a kind of Recurrent Neural Network (RNN). Its performance to recognize characters is made in real-time, where the images collected from the cameras are sent to the data center, processed and then used as input to Tesseract that will detect the license plate characters.

### 2.3. Related Works

The number of vehicles has been increasing in the last few years, and the ALPR has become a very important tool that helps in the monitoring and control of the vehicles. However, the existing diversity of plate formats, different scales, rotations, and no uniform illumination conditions during capturing the image [5] have become a big issue for the ALPR system. There are several methods and researches about ALPR. However, most of them are not applied in real-time. In this subsection, the related works are highlighted.

In [21], a project whose main objective is the improvement of the digits and features representations through Deep Learning techniques is presented. The authors developed a dataset composed of approximately 2000 images that are divided into a training set and test set. The authors conclude that the data augmentation techniques and the increase in locally connected layers have greatly increased the accuracy obtained with the datasets used in their approach.

In [22], the authors introduced a character recognition algorithm based on supervised classification. The authors make a description of the character classes using model the pixel sequence behavior in texts present in images. To reach the results, the strategy used is to describe the characters framed in a mesh, observing the transitions between the levels of gray from pixels, therewith, the pixel behavior in each class was determined, what could be used to classify other records. The authors concluded that the proposed algorithm in their project has obtained reasonable results for tasks that require real-time digit identification even with handwriting characters, recognizing 92,336 characters per minute.

In [23], a system was presented for the recognition of numerical digits in license plates. For the position recognition of the license plate, the White Top-Hat Transform was used so that the bright areas over dark objects could be removed and the image brightness could be corrected, soon after, the binarization process was done to transform it in black and white. After the localization of the license plate, a character’s segmentation in a horizontal and vertical projection of the plate was made and the numbers were transformed into vectors. Finally, a multilayer perceptron network was resorted to for character recognition. According to [23], the classifier identified 37 out of 43, achieving an accuracy of 76%.

In [24], an approach was proposed to detect license plates in varying illumination conditions. A binarization method was used on the pre-processing step for plate segmentation, and the thresholding method was applied to the image. However, the experiment environment and processing time were not mentioned in the paper. In [25], a pre-processing and segmentation method for number plate localization was used. In the pre-processing, a global threshold was used to map the color intensity into a grayscale. The authors assumed two points: plates are oriented horizontally and there is a significant intensity difference between plate background and character foreground. However, in a real environment, these assumptions can change.

## 3. The Propose Vehicular Identification System Based on OCR for ITS

In this section, we introduce the algorithm developed to make the plate license recognition.

### 3.1. System Overview

**Figure 3:**
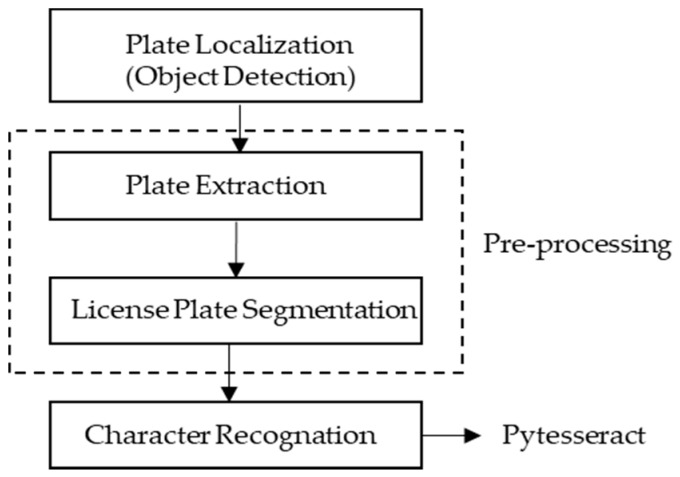
Flowchart of the proposed algorithm.

The proposed system aims to read frames of a camera and recognize the characters present on license plates in real-time. Figure 3 shows the flowchart of the proposed algorithm.

When SPAN detects a vehicle, the proposed algorithm starts the plate localization function to extract the license plate from the vehicle image. This is made by recognizing rectangular objects on the vehicle image. When the proposed algorithm finds a plate on the vehicle image, it extracts the plate area creating a new image. Once the plate image is extracted, it starts the pre-processing phase of the image. The pre-processing is necessary due to variations of the environment (illumination, background, texture) and is aimed at reducing possible noises on the image. It is used in the proposed system as a bilateral filter [26] to smooth the image and reduce the noise. After the pre-processing phase, the proposed algorithm uses the Tesseract [20] to make the recognition of the characters present on the plate image. The pseudo-code of the proposed algorithm is shown in Section 3.2 below.

### 3.2. The Algorithm

**Figure 4:**
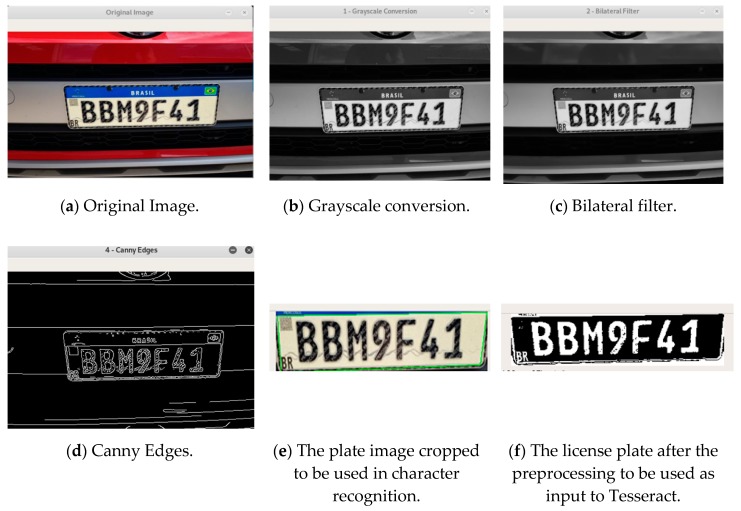
The proposed algorithm applied to the image to detect the characters. (**a**) Original Image, (**b**) Grayscale conversion, (**c**) Bilateral filter applied, (**d**) Edges detection, (**e**) The license plate cropped from the main frame, (**f**) The license plate after the treatment.

In this subsection, how the proposed algorithm works is described in Algorithm 1, since the plate image detection until the character recognition. The proposed algorithm is described below, and Figure 4 [27] shows the results of the proposed algorithm using the images taken from the sensors.
**Algorithm 1:** The proposed algorithm1. Start Video Capture 2. Read Video_Frame 3. Resize Video_Frame 4. Gray_Frame = Transform to grayscale (Video_Frame) 5. Apply filter to remove the noise (Gray_Frame) 6. Frame_Edges = Detect edges (Gray_Frame) 7. Frame_Contours = Find contours (Frame_Edges) 8. Sort (Frame_Contours) 9. Declare Number_Plate_Contour 10. Declare Largest_Rectangle 11. **for** Contour **in** Frame_Contours **do**
12.    Perimeter_Rectangle = Calculate perimeter (Contour) 13.    Approx_Rectangle = Find the approximate rectangle (Perimeter_Rectangle) 14.    **if** (length (Approx_Rectangle) == 4) **then**
15.      Number_Plate_Contour = Approx_Rectangle 16.      Largest_Rectangle = Find area (Approx_Rectangle) 17.      **break**
18. *x*,*y*,*w*,*h* = Calculate up-right bounding rectangle (Largest_Rectangle) 19. Cropped_Frame = Crop using x,y,w,h (Video_Frame) 20. Draw Largest_Rectangle contours on Video_Frame 21. Transform to grayscale (Cropped_Frame) 22. Frame_Threshold = Binarize (Cropped_Frame) 23. Kernel = New square object of size 1x1 24. Image_Dilation = Dilates using Kernel (Frame_Threshold) 25. Dilated_Image_Contours = Find contours (Image_Dilation) 26. Sorted_Dilated_Contours = Sort (Dilated_Image_Contours) 27. **for** Dilated_Contour **in** Sorted_Dilated_Contours 28.    x,y,w,h = Calculate up-right bounding rectangle (Dilated_Contour) 29.    Draw a rectangle of dimensions x,y,w,h on Video_Frame 30. **end for**
31. Transform to binary (Gray_Frame) 32. License_Plate_Characters = Transform to string (Gray_Frame) 33. **if** length(License_Plate_Characters) > 0 **then**
34.    Get License_Plate_Characters 35. **end if**
36. **return** Video_Frame

As can be seen in the proposed algorithm, in the first line the video camera is started. So, the video camera reads and resizes the frames (lines 2, 3). The frames are resized following the distance of the camera to the vehicle to have a better perspective of the plate. From line 4 to line 7, filters are used to find the contours of the image. For this purpose, firstly a grayscale filter is applied in the image as illustrated in Figure 4b. After that, a bilateral filter is applied to remove noises on the image as shown in Figure 4c. So, the noises could be removed without damaging the edges of the frame, and the edges are detected as shown in Figure 4d. A loop starts at line 11 to find the rectangle that has the plate numbers. Once found, this rectangle is cropped as illustrated in Figure 4e (line 19 of the algorithm). The block that includes the lines 21 to 31 exposes the pre-processing of the cropped image of the main frame. Figure 4f represents the result of this prepossessing. Finally, the characters are read at line 34 of the algorithm.

## 4. Evaluation Scenario and Performance Measurements

**Figure 5:**
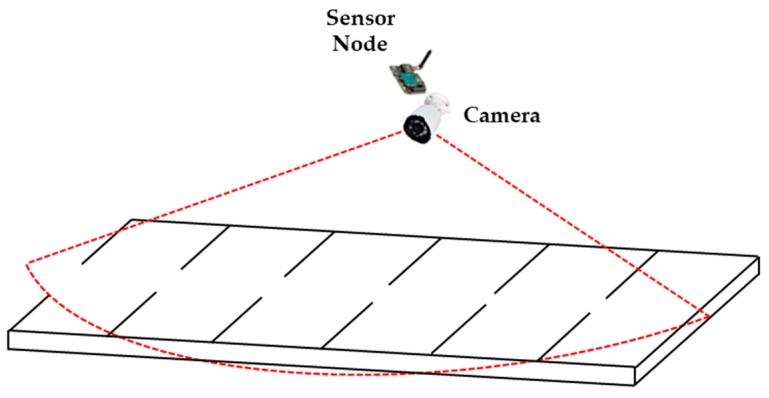
Evaluation scenario.

The proposed system is implemented in Python. The hardware used is an Alienware, Intel Core I7, 16 GB of RAM, 1 TB of HD. The Tesseract version used is the 4.0 [20]. The proposed algorithm is evaluated in real-time using real parking. Figure 5 shows the evaluation scenario, where the red dotted lines show the vision angle of the camera regarding the parking slots. Every time a car arrives, the camera takes a picture of the vehicle and sends the picture to the Data Center that runs the proposed algorithm to find the plate characters as described in the proposed algorithm in Section 3.2.

The performance of the proposed algorithm is measured by metrics derived from the confusion matrix [28,29]. The recognition of a license plate can only be considered correct if all characters that compose the license plate are correctly recognized. There are some cases where the license plate is incorrectly recognized (one or more characters are incorrectly recognized), or it is not recognized (any character is recognized). In this case, we use the generalized confusion matrix for multiple classes [30] to verify the performance of the proposed solution, where its class corresponds to a character. Table 1 shows the confusion matrix with *n* classes.

Each class showed in Table 1 corresponds to a character used to compose the license plate. In this work, the following characters: “0”, “1”, “2”, “3”, “4”,”5”, “6”, “7”, “8”, “9”, “A”, “B”, “C”, “D”, “E”, “F”, “G”, “H”, “I”, “J”, “K”, “L”, “M”, “N”, “O”, “P”, “Q”, “R”, “S”, “T”, “U”, “V”, “X”, “W”, “Y”, “Z” are considered. Considering the confusion matrix with *n* classes showed in Table 1, the total number of True Negative (TN), True Positive (TP), False Positive (FP) and False Negative (FN) for each class *n* are calculated as follows:

(1) $$Total\_FN_{i}\;=\;\overset{n}{\underset{\begin{array}{c} J=1\\ J\neq i \end{array}}{\sum }}x_{ij}$$

(2) $$Total\_FP_{i}\;=\;\overset{n}{\underset{\begin{array}{c} J=1\\ J\neq i \end{array}}{\sum }}x_{ij}$$

(3) $$Total\_TN_{i}\;=\;\overset{n}{\underset{\begin{array}{c} J=1\\ J\neq i \end{array}}{\sum }}\;\times \;\overset{n}{\underset{\begin{array}{c} K=1\\ K\neq i \end{array}}{\sum }}x_{jk}$$

(4) $$Total\_TP_{all}\;=\;\overset{n}{\underset{\begin{array}{c} J=1 \end{array}}{\sum }}x_{jj}$$

Considering the generalized confusion matrix, the overall accuracy is calculated in accordance to Formula (5):

(5) $$\mathrm{Overall}\;\mathrm{Accuracy}\;=\;\frac{Total\_TP_{all}}{Total\;Number\;of\;Testing\;Entries}$$

The processing time measurement represents the time spent processing the image to recognize the characters and store the plate numbers in the database, where this information is available to be accessed by the web application. Moreover, the processing time is calculated taking into account the environment illumination.

## 5. Numerical Results

**Figure 6:**
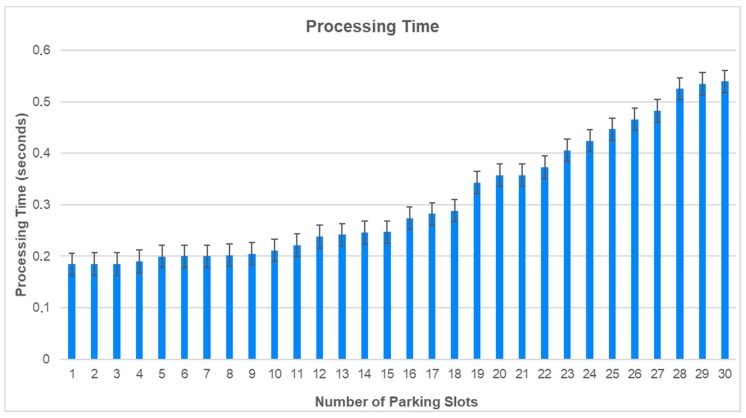
Processing Time.

In this section, we present the numerical results of the proposed algorithm. Every time a car arrives, the camera takes a picture and sends the picture to the Data Center that runs the proposed algorithm as described in the proposed algorithm in Section 3.2. To verify the performance of the proposed algorithm, the experiments were carried out at different times of the day in the morning, afternoon and at night. So it is possible to evaluate the proposed algorithm in the different environments of elimination. Figure 6 shows the processing time to recognize the plate numbers.

**Figure 7:**
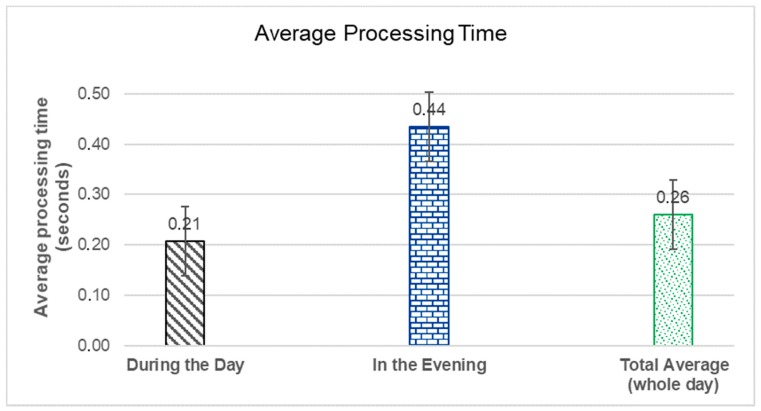
Average Processing Time.

The processing time starting from slot 1 to slot 18 was detected during the day (in the morning and on the afternoon), and the processing time from slot 19 to 30 was detected when the illumination started to get dark, and in this case, it used the parking illumination (in the evening). Figure 7 shows the average processing time.

As can be seen in Figure 7, during the day the processing average of the processing time is 0.21 s, and in the evening, the processing time is 0.44 s. The processing time increases in the evening because the illumination in the evening changes, becoming darker. However, the processing time, even in the evening, is low. Considering the illumination in both cases, it is possible to see that the average of the processing time during the whole day is 0.26 s, which is acceptable. 

**Figure 8:**
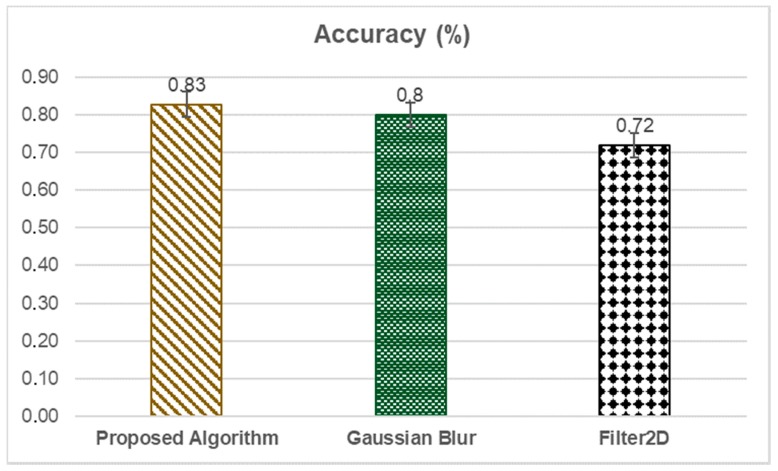
Accuracy of the proposed algorithm and the used filters.

Figure 8 shows the accuracy of the proposed algorithm. Due to the variation of the environment illumination, we carried out the proposed algorithm changing the used filters. So, we also used the Gaussian Blur filter and the Filter 2D.

As seen in Figure 8, the accuracy of the proposed algorithm is 83%, and the accuracy of the filters Gaussian Blur and Filter 2D is 80% and 72% respectively. So, the proposed algorithm shows a good result considering the filters Gaussian Blur and Filter 2D.

## 6. Conclusions

In this paper, a system that identifies in real-time vehicle license plate characters using optical character recognition (OCR) was proposed. The proposed system makes use of the intelligent parking service (SPANS) which is a framework used to detect available parking slots using computer vision functions. The proposed system uses the camera of the SPANS to get the images and information of the parking slots. Once a vehicle is detected, the proposed system takes a picture of the vehicle and uses this image to identify the license plate number of the vehicle. So, the identified number is stored on the system, and this information can be made available to public agencies such as traffic departments.

The algorithm used by the proposed system to detect the plate and to recognize the plate characters was described. The description starts with the plate image detection until the character recognition. Experiments were carried out at different times of the day. So, it was possible to evaluate the proposed system to take into account the variations in the environment illumination. So, different image filters were used in the proposed system, and the most robust filter was chosen to integrate the system. 

Due to the variation of the environment illumination, the proposed system was evaluated using different filters on the pre-processing phase. The filters tested were the Gaussian Blur filter and the Filter 2D. The results showed that the proposed system had better performance when the bidirectional filter was used, detecting the plate characters on the environment with illumination variations. The processing time to make the plate recognition is low, and the proposed system showed a good performance in a real environment. However, it was possible to verify that the character recognition is sensitive to the environment illumination, and to improve the accuracy, new experiments will be done considering other types of filters as well as new deep learning methods will be implemented in the future works.

## Figures and Tables

**Table 1:** Confusion matrix with *n* classes.

		Predicted Character					
		Class 1	Class 2	Class 3	Class 4	…	Class *n*
**Actual Character**	**Class 1**	*x*_11_	*x*_12_	*x*_13_	*x*_14_	…	*x*_1*n*_
	**Class 2**	*x*_21_	*x*_22_	*x*_23_	*x*_24_	…	*x*_2*n*_
	**Class 3**	*x*_31_	*x*_32_	*x*_33_	*x*_33_	…	*x*_3*n*_
	**Class 4**	*x*_41_	*x*_42_	*x*_43_	*x*_44_	…	*x*_4*n*_
	**…**	…	…	…	…	…	…
	**Class *n***	*x_n_*_1_	*x_n_*_2_	*x_n_*_3_	*x_n_*_4_		*x_nn_*
